# Specific Oncogenic Activity of the Src-Family Tyrosine Kinase c-Yes in Colon Carcinoma Cells

**DOI:** 10.1371/journal.pone.0017237

**Published:** 2011-02-24

**Authors:** Florence Sancier, Aurélie Dumont, Audrey Sirvent, Ludmilla Paquay de Plater, Thomas Edmonds, Géraldine David, Michel Jan, Catherine de Montrion, Francis Cogé, Stéphane Léonce, Michael Burbridge, Alain Bruno, Jean A. Boutin, Brian Lockhart, Serge Roche, Francisco Cruzalegui

**Affiliations:** 1 Institut de Recherches Servier, Croissy-sur-Seine, France; 2 Equipe labellisée LA LIGUE 2009, Centre de Recherche de Biochimie Macromoléculaire, UMR5237 Centre National de la Recherche Scientifique et Université de Montpellier, Montpellier, France; The Beatson Institute for Cancer Research, United Kingdom

## Abstract

c-Yes, a member of the Src tyrosine kinase family, is found highly activated in colon carcinoma but its importance relative to c-Src has remained unclear. Here we show that, in HT29 colon carcinoma cells, silencing of c-Yes, but not of c-Src, selectively leads to an increase of cell clustering associated with a localisation of β-catenin at cell membranes and a reduction of expression of β-catenin target genes. c-Yes silencing induced an increase in apoptosis, inhibition of growth in soft-agar and in mouse xenografts, inhibition of cell migration and loss of the capacity to generate liver metastases in mice. Re-introduction of c-Yes, but not c -Src, restores transforming properties of c-Yes depleted cells. Moreover, we found that c-Yes kinase activity is required for its role in β-catenin localisation and growth in soft agar, whereas kinase activity is dispensable for its role in cell migration. We conclude that c-Yes regulates specific oncogenic signalling pathways important for colon cancer progression that is not shared with c-Src.

## Introduction

Cytoplasmic tyrosine kinases of the Src family (SFKs) play important roles in signal transduction induced by a large number of extracellular stimuli including growth factors and integrins [1], [2]. They include 8 cellular members, c-Src, Fyn and c-Yes being widely expressed. SFKs contain a unique N-terminus with a myristoylation site required for membrane localisation, SH3 and SH2 domains used for protein-protein interactions, a catalytic domain and a C-terminal Tyr residue, that when phosphorylated by Csk, inhibits kinase activity [3]. SFKs also exhibit oncogenic activity when deregulated, a situation frequently observed in human cancer. Remarkably, elevated SFK activity is found in more than 80% of human colorectal cancer (CRC) and this has been associated with poor clinical outcome [4]. c-Src deregulation is thought to be an important event for colon tumorigenesis tumour growth, angiogenesis and metastasis [4]. Therefore, c-Src is an attractive therapeutic target and several small molecule inhibitors are currently being tested in clinical trials [5].

The SFK member c-Yes is the cellular counterpart of the viral v-Yes protein encoded by the Yamaguchi avian sarcoma virus [6]. c-Yes exhibits the highest homology with c-Src among SFK members, with 70% identity outside the N-terminus. As in v-Src, a C-terminal truncation in v-Yes removes the negative regulatory Tyr allowing the kinase to be constitutively active and highly oncogenic. While such activating mechanism has not been reported in human cancer, c-Yes is found frequently activated in CRC. Remarkably, c-Yes activation in CRC correlates more closely with poor prognosis [7], [8] than does c-Src activation. Despite the above evidence suggesting a role for c-Yes in CRC, functional data supporting this notion are missing. The majority of data published on SFKs has focused on c-Src and it has been generally accepted that c-Yes may be redundant in malignancies. This idea has been supported by gene knock-out experiments in mice and corresponding embryonic fibroblasts, which pointed to partial redundant functions during embryogenesis [9] and cellular division [10]. The major structural difference between these SFKs lies in the unique N-terminus with additional palmitoylation site present in c-Yes and absent in c-Src [1]. This post-translational modification stabilises c-Yes in specific sub-cellular compartments, including cholesterol-enriched membrane domains present at tight and adherent junctions [11]. Due to the absence of such lipid attachment, c-Src shows higher mobility at membranes and therefore is localised at focal adhesions [12]. Thus, membrane partitioning may contribute to unique signalling emanating from these SFKs.

Several lines of evidence point to selective functions for c-Yes in cellular signalling leading to transcytosis and cell-cell adhesions [13]. For instance, c-Yes, and not c-Src is reported to form a calcium-dependent complex with occludin at tight junctions [14]. A role of c-Yes in adherens junctions has been revealed in yes-/- mice. Although no obvious phenotype was initially observed [9], it was found that deletion of c-Yes led to decreased vascular permeability leading to lower extravasation of tumour cells and reduced leakeage during ischemia [15]. It has also been reported that pharmacological inhibition of SFKs with pan-SFK compounds increases cell-cell adhesion via an elevation of E-cadherin and VE-cadherin in epithelial or in endothelial cell-cell junctions respectively [16], [17], [18], [19]. Whether c-Src or c-Yes has a selective role in cell-cell adhesion of tumour cells and more generally in carcinogenesis is largely unknown. Here we have used an RNA interference approach to address the specific function of c-Yes in CRC cells. We show in HT29 cells that, despite high endogenous levels of deregulated c-Src activity, c-Yes drives selective oncogenic signalling required for transformed phenotype of these cancer cells including cell-cell adhesion, growth/survival and invasion.

## Results

### Knock-down of c-Yes results in cell clustering of colon cancer cells

**Figure 1:**
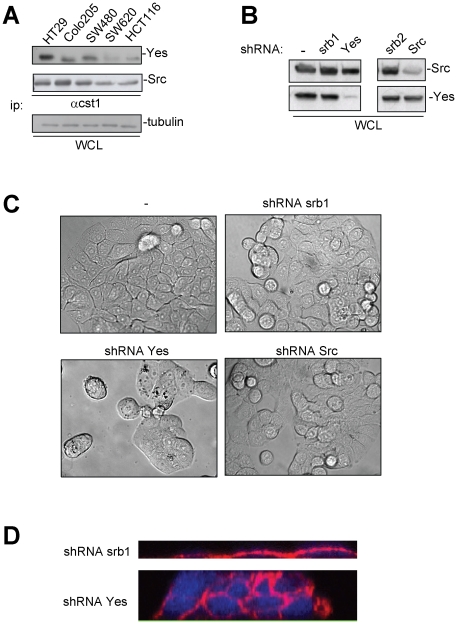
Stable c-Yes but not c-Src knock-down results in cell clusters. (A) Western blots showing c-Yes and c-Src proteins in a lysate (WCL) of CRC cell-lines. (B) c-Yes and c-Src proteins in lysates of HT29 cells expressing shRNA constructs. Srb1 shRNA is a control shRNA and srb2 a control version of c-Src lentiviral shRNA. (C) DIC microscopy images showing the morphology of HT29 cells expressing shRNAs. (D) Confocal microscopy reconstitution HT29 cells expressing shRNA and stained with β-catenin antibodies.

We first measured c-Yes expression in various CRC cells-lines. HT29 was found to express the highest amount of c-Yes among cells tested (Fig. 1A), therefore this cell-line was used to address c-Yes oncogenic signalling in CRC cells. A comparison of c-Src and c-Yes mRNA levels indicated that these cells contain 3.8-times higher c-Src levels, suggesting that c-Yes represents at most, 25% of the SFK pool in HT29 cells (Fig. S1A). The selective function of c-Yes was next addressed by depleting c-Yes levels by the use of a specific shRNA. c-Yes protein expression was reduced by 85–90% (Fig. 1B and Fig. S1), without affecting c-Src expression. Cellular infection with retroviruses expressing shRNA targeting c-Src gave a milder reduction in Src expression (70–75%) without affecting c-Yes level (Fig. 1B and Fig. S1). Since it has been shown previously that pharmacological inhibition of SFKs results in formation of cell clusters [17], [19], [20] we examined the formation of clusters by Differential Interference Contrast microscopy. Interestingly, c-Yes knock-down was sufficient to induce formation of cell clusters (Fig. 1C). Confocal microscopy revealed that c-Yes knock-down-induced clusters containing up to five layers of cells (Fig. 1D). Similar effects were observed when silencing c-Yes with transient transfection of individual siRNA sequences (Fig. S2), indicating that these inhibitory effects were not due to off-target and/or long-term depletion of c-Yes. In contrast, c-Src knock-down resulted in a mild effect on the morphology of the cell monolayer (Fig. 1C and Fig. S2C), suggesting that the role of c-Yes on this cellular process is not shared by c-Src. Besides c-Src and c-Yes, Lyn, but not Fyn, is also detected in HT29 cells. We then examined the role of Lyn in cell adhesions with a similar siRNA strategy. Although Lyn knock-down was very efficient, there was no impact on cell morphology (Fig. S3B). Taken together, these data point to a selective role of c-Yes in the modulation of HT29 cell adhesive properties.

### c-Yes knock-down strengthens β-catenin accumulation at cell-cell junctions

**Figure 2:**
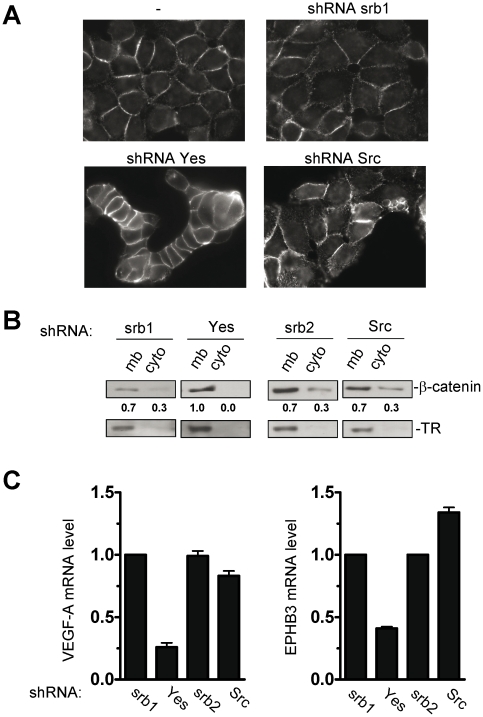
β-catenin accumulates at cell junctions and reduces expression of β-catenin target genes in c-Yes knock-down cells. (A) Confocal microscopy images of β-catenin in HT29 cells expressing indicated shRNAs. (B) Western blot analysis showing the level of β-catenin in membrane (mb) and cytosolic (cyto) fractions from cells expressing indicated shRNAs. The level of Transferrin Receptor (TR) is also shown and was used as a control of membrane fractions. (C) VEGF-A and EphB3 quantitative RT-PCR in cells expressing indicated shRNA. RNA is expressed relative to expression in srb1 or srb2 control shRNA expressing cells.

We next investigated the effect of c-Yes depletion on cell clustering in further detail. SFKs, and in particular c-Src, have been described as important regulators of focal adhesions via recruitment by FAK auto-phosphorylated on Tyr397. This recruitment leads to phosphorylation of FAK at tyrosines 861 and 925 by SFKs leading to focal adhesion plasticity [21]. FAK phosphorylation at Tyr861 was unaffected by c-Yes knock-down, in contrast to c-Src knock-down (Fig. S2B). Accordingly, FAK silencing failed to induce similar cell clusters (Fig. S3A), suggesting that the morphological effect induced by c-Yes depletion is not due to a defect on cell spreading. Since localisation of β-catenin at the membrane has been reported to be in part regulated by SFKs [17], [20], we examined its localisation. In HT29 cells, β-catenin is present in majority at cell-cell junctions and the cytoplasm. Using confocal microscopy we observed that c-Yes knock-down induced a strong accumulation of β-catenin and E-Cadherin at the cell-cell junctions (Fig. 2A and S4). Biochemical analysis of β-catenin cytosolic and membrane fractions tightly correlated with these observations (Fig. 2B). In contrast, c-Src knock-down had only a mild effect on β-catenin membrane accumulation (Fig. 2A and B). This suggests that cell clustering induced by c-Yes depletion primarily relies on a modulation of adherent junctions.

### c-Yes knock-down affects β-catenin signalling in colon cancer cells

We next examined whether β-catenin accumulation at cell-cell junction has functional consequences on its ability to drive transcription of its cognate genes. Amongst its target genes, VEGF-A and EphB3 have demonstrated to be highly sensitive to a reduction of β-catenin activity [22]. As shown in figure 2C, a 60% reduction in VEGF-A and EphB3 mRNA levels was observed upon c-Yes depletion. Accordingly, confocal microscopy analysis revealed some nuclear β-catenin exclusion upon c-Yes depletion (Fig. S4). However no such a change in nuclear β-catenin level could be obtained by biochemical analysis, probably because of its low abundance in nuclear compartments of HT29 cells and the low sensitivity of the biochemical approach as compared to confocal microscopy analysis (not shown). Interestingly, no significant reduction in VEGF-A or EphB3 mRNA was observed in c-Src depleted cells, showing specificity among Src members (Fig. 2C).

### c-Yes silencing leads to increased apoptosis and reduced tumour growth

**Figure 3:**
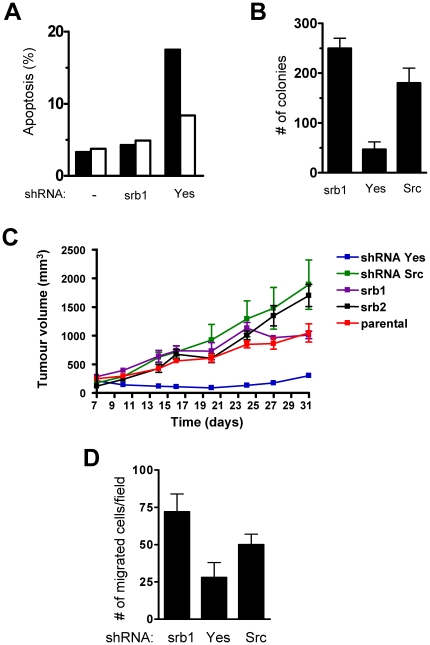
c-Yes knock-down increases apoptosis and decreases growth in soft-agar and in mouse xenografts. (A). Percentage of apoptotic cells obtained after stable transduction of indicated shRNA and revealed by Annexin V (black box) and propidium idodide (white box). (B) c-Yes depletion inhibits cell growth in soft-agar. Number of colonies obtained from HT29 cells expressing indicated shRNA grown in soft-agar conditions. The mean ± SD (n>3) is shown. (C) c-Yes depletion inhibits subcutaneous tumour growth in nude mice. Cells expressing indicated shRNA were implanted subcutaneously in five SCID mice. (D) c-Yes depletion inhibits cell migration. Statistical analysis of the number of HT29 cells expressing indicated shRNA/field that have migrated in Boyden chamber assays in the presence of EGF (20 ng/ml). The mean ± SD (n>3) is shown.

We next analysed the consequences of c-Yes knock-down on cell growth and survival. We first observed that c-Yes specific depletion consistently showed a doubling time 1.4 fold longer than control HT29 cells (Fig. S5B). We measured the distribution of cells at each phase of the cell cycle by flow cytometry and observed only 1.6-fold increase in the fraction of cells in G2/M when compared to cells expressing a scramble version of c-Yes shRNA (srb1), consistent with an important role of SFK in cell division [10] (Fig. S5A). Our cytometry experiments also showed a 4 to 6 fold increase in sub-G1 cells in c-Yes depleted cells (Fig. S5A). Consistent with these observations, these cells showed an increase in apoptosis as revealed by annexin V labelling (Fig. 3A) and caspase activity (>1000 fold as compared to control cells) (Fig. S5C). We examined further the effect of c-Yes on cell growth/survival by studying the potential of cells for substrate-independent growth. We found that c-Yes silencing induced 85% decrease in the number of colonies grown in soft agar (Fig. 3B). Moreover, colonies were smaller in size when compared to the ones obtained with control cells (not shown). Again, c-Src depletion induced a milder effect. We next wished to confirm this data *in vivo*, by implanting these cells sub-cutaneously into SCID mice. In contrast to control or c-Src knock-down cells, c-Yes-deficient cells failed to produce large tumors (Fig. 3C). Western blotting of tissue extracts confirmed that the small masses generated by these cells had reduced c-Yes levels (Fig. S6). We concluded to an important function for c-Yes in growth/survival of HT29 cells.

### c-Yes silencing reduces cancer cell migration and liver metastasis in nude mice

We next evaluated the impact of c-Yes knock-down on cell invasion. c-Yes knock-down reduced cell migration by 75% in a Boyden chamber assay in vitro, whereas c-Src knock-down gave a milder effect (Fig. 3D). Accordingly, c-Yes silencing also affected the capacity of cells to invade liver tissue in an experimental metastasis assay. Strikingly, none of the mice injected with c-Yes depleted cells showed signs of liver metastasis in contrast to control cells that led to the macroscopically visible metastasis (>2 mm) in the livers of all mice tested (Table 1). Again, c-Src knock-down resulted in a moderate reduction in the extent of liver metastases (Table 1). These results indicate that c-Yes play important roles in the metastatic capacity of colon carcinoma cells.

**Table 1:** c-Yes knock-down inhibits liver metastasis after intrasplenic injection.

	Metastatic index	
HT29 cells	Micro-metastases (<2 mm)	Metastases (>2 mm)
**parental**	0.87	3
**srbl**	1	2.5
**c-Yes**	0.12	0
**c-Src**	0.66	2

Cell expressing indicated shRNA were injected in the spleen of SCID mice and livers analysed 30 days after surgery. Metastases (>2 mm) and micrometastases (<2 mm) were scored according to the relative extent of invaded tissue and a metastatic index calculated as described in Material and Methods is shown.

### c-Yes oncogenic signalling is not shared with c-Src

**Figure 4:**
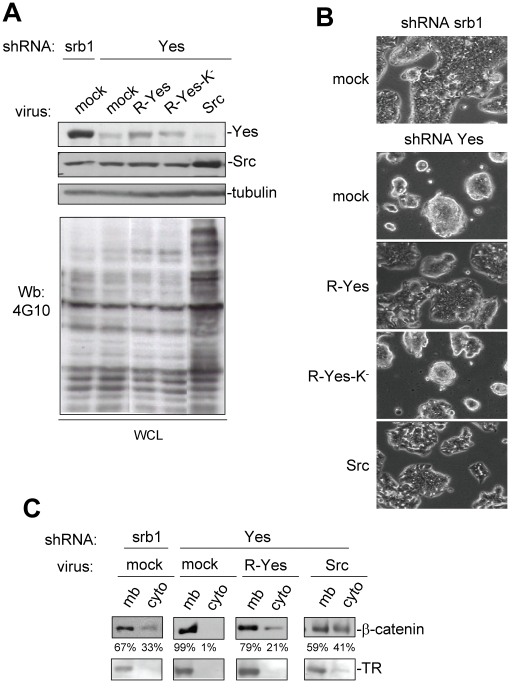
High expression of c-Src can rescue cell adhesive properties and cytosolic β-catenin of c-Yes depleted cells. (A). Western blotting showing the level of c-Src, c-Yes, phosphotyrosine content (4G10) and tubulin as a control of HT29 cells expressing indicated shRNA that were infected with control virus (mock) or viruses expressing indicated SFK construct. (B) Restoration of cell morphology by c-Yes or c-Src re-introduction. Cell morphology of c-Yes depleted HT29 cells that were infected with control virus (mock) or viruses expressing indicated SFK construct. (C) Restoration of β-catenin localisation by c-Yes and c-Src expression. Western blot analysis showing the level of β-catenin in membrane (mb) and cytosolic (cyto) fractions from cells expressing indicated shRNA and infected with indicated virus. The level of Transferrin Receptor (TR) is also shown and was used as a control of membrane fractions.

**Figure 5:**
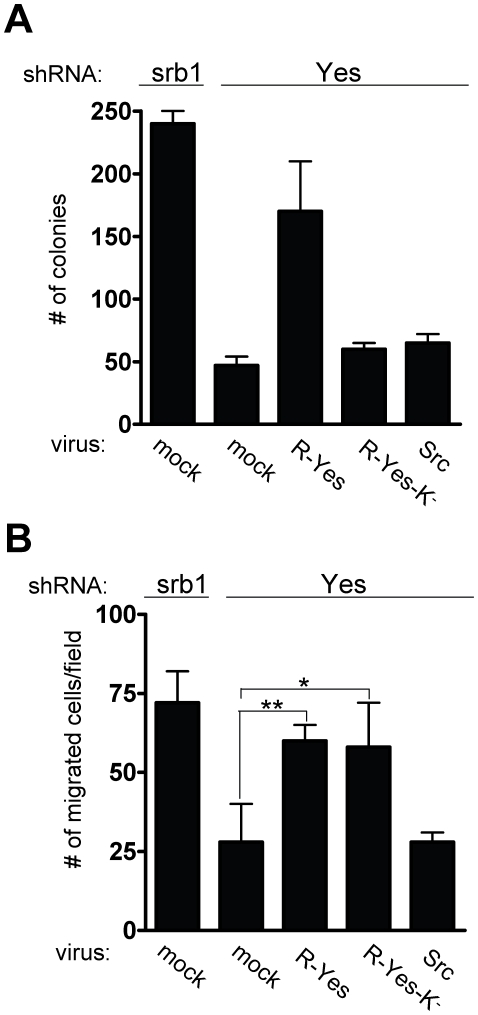
c-Src expression does not restore transforming properties of c-Yes depleted cells. (A) c-Src expression does not restore cell growth in soft-agar of c-Yes depleted cells. Number of colonies obtained from HT29 cells expressing indicated shRNA and infected with shown retroviruses. The mean ± SD (n>3) is shown. (B) c-Src expression does not restore cell migration of c-Yes depleted cells. (B) Statistical analysis of the number of indicated HT29 cells/field that have migrated in Boyden chamber assays in the presence of EGF (20 ng/ml). The mean ± SD (n>3) is shown. * *P*<0.05 and ** *P*<0.01 using a student's t-test.

Finally, we analysed the capacity of c-Src to reverse the phenotypes of c-Yes depleted cells. c-Src or an shRNA-resistant version of c-Yes (R-Yes) was transduced into c-Yes depleted cells by retroviral infection. c-Yes levels were restored by 50% and c-Src levels were increased by >3-fold above endogenous c-Src levels (Fig. 4A). Ectopic c-Src was very active in these cells as shown by the increase in global tyrosine phosphorylation. In contrast, partial restoration of c-Yes levels only poorly affected global tyrosine phosphorylation (Fig. 4A). Nevertheless, stable introduction of the R-Yes construct was sufficient to reverse the clustering phenotype of c-Yes knock-down cells, (Fig. 4B). This effect was dependent upon the catalytic activity of c-Yes as it was not observed by the expression of the kinase-defective R-Yes-K^−^ construct. In agreement with this notion, we also observed that the increase of β-catenin membrane localisation due to c-Yes silencing can be restored by R-Yes. Immunofluorescence experiments confirmed the restoration of intracellular β-catenin by expression of R-Yes (Fig. S7). Interestingly, R-Yes-K^−^ was unable to show this effect confirming c-Yes kinase-dependent signalling towards β-catenin and cell-cell adhesion (supplementary Fig. S7). Interestingly, we found that in our conditions, ectopic c-Src also reversed the clustering phenotype of c-Yes knock-down cells to some extent and restored cytosolic β-catenin (Fig. 4B and C). This effect may be explained by the relative high level of expressed c-Src to endogenous c-Yes (10-15 fold), so that signalling specificity is lost. Moreover, this data indicates that c-Yes signalling specificity can be overcome by the expression of a large excess of c-Src. Finally, we wished to address whether c-Src shares c-Yes oncogenic activities in these CRC cells. Interestingly, c-Src overexpression neither restored anchorage-independent growth nor cell migration of c-Yes depleted cells, unlike R-Yes (Fig. 5A and B). Again, the effect of c-Yes on cell growth was kinase-dependent as it was not observed with R-Yes-K^−^ (Fig. 5A). Surprisingly, a different situation was observed on cell migration. While the R-Yes construct selectively restored cell migration of c-Yes-depleted HT29 cells, R-Yes-K^−^ also restored this biological property (Fig. 5B). This suggests that c-Yes migratory signalling is not dependent upon its kinase activity, unlike cell growth.

## Discussion

In this paper we show that in HT29 cells, down-regulation of c-Yes levels is sufficient to induce epithelial characteristics such as elevated localisation of β-catenin at cell-cell-adhesions. c-Yes knock-down also leads to an increase in apoptosis, reduced substrate-independent growth, loss of the capacity to form sub-cutaneous tumors in nude mice and to invade tissue in an experimental metastasis model. Similar results have been obtained in additional CRC cells tested, indicating that this oncogenic function of c-Yes is not specific to the cell-line used in this study (F. Dubois and S. Roche, unpublished data). Our data uncover for the first time a unique function for c-Yes in β-catenin signalling and tumorigenicity of CRC cells. Moreover, our results show a milder inhibitory effect following c-Src silencing, suggesting that c-Yes could be a more important player than c-Src in CRC tumorigenesis. Nevertheless, it remains possible that the low effect of c-Src knock-down described here is due to residual deregulated c-Src activity in HT29 cells being sufficient to maintain a transformed phenotype. Based on the relative expression of c-Src and c-Yes in parental cells and the level of knock-down achieved, we deduced that the residual c-Src mRNA copy number achieved with our shRNA strategy is comparable to the parental cell c-Yes mRNA copy number (Figure S1A). Therefore, a shRNA approach targeting c-Src levels more efficiently may address this issue unambiguously. Nevertheless, c-Src silencing obtained with our strategy was sufficient to reduce invasion *in vivo* approximately by half, while having no effect on subcutaneous tumour growth. This suggests that CRC metastatic invasion is more sensitive to c-Src knock-down than primary tumour growth, thus predicting a selective therapeutic effect of SFK inhibitors on metastasis.

Our data show that c-Yes transforming activity is not redundant to that of c-Src, although the latter is predominantly expressed in these tumours. The exact selective mechanism downstream of c-Yes is an important issue that needs to be addressed. In this regard, we believe that one important mechanism may rely on specific subcellular localisation of SFK family members, leading to phosphorylation of specific substrates. This hypothesis is sustained by the specific localisation of c-Yes in cholesterol-enriched microdomains found at adherent and tight junctions, probably due to an additional palmitoylation site present in c-Yes. Accordingly, several labs including ours have shown that this membrane partitioning regulates SFK signalling specificity leading to mitogenesis [23] and neoplastic transformation [12], [24]. An additional mechanism for selective signalling may involve substrates that interact with the unique, SH3 or SH2 domains of these SFK. The nature of these c-Yes partners in CRC is currently unknown, but some obvious candidates include components of the adherens junctions such as E-cadherin and β-catenin. Accordingly, our report suggests that one mechanism by which c-Yes regulates its oncogenic activity is by modulation of β-catenin subcellular localisation at the adherens junctions counteracting its nuclear transcriptional activity, this cellular process being regulated by tyrosine phosphorylation. Interestingly, the fact that c-Src can restore cytosolic β-catenin while having no effect on cell transforming activities of c-Yes depleted cells suggests that c-Yes may induce additional signalling pathways to ensure neoplastic properties of CRC cells. A phospho-proteomic analysis uncovering c-Yes selective substrates is under current investigation to address this issue.

Finally, our data also reveal an unanticipated role of c-Yes in migration independent of its catalytic activity. An adapter function of SFK has been already documented with the example of c-Src during integrin signalling leading to cell spreading [1]. Therefore, we anticipate a similar adaptor function for c-Yes, implicating specific c-Yes SH2/SH3 binders to be identified. This observation may have important implication in cancer therapy involving SFK catalytic inhibitors and may explain, at least in part, the discrepancy observed between the dramatic loss of tumorigenicity in c-Yes-depleted cells compared to mild effects of SFK kinase inhibitors in vivo. Alternatively, pan-SFK pharmacological inhibitors could affect the activity of other kinase or non-kinase targets that may counter-act the effect of selectively inhibiting c-Yes inhibition. Given the difficulty in obtaining specific c-Yes inhibitors, therapeutic siRNAs would be necessary to test this hypothesis. In conclusion, these data would predict therapeutic value of siRNA targeting c-Yes in CRC and confirm SFKs as attractive therapeutic targets in this cancer.

## Materials and Methods

### 2.1 Reagents

[γ^32^P]ATP was purchased from Amersham. Epithelial Growth Factor, Hexadimethrine Bromide, G418 and Puromycin from Sigma Aldrich (St Quentin, France). SFK antibodies (cst1) were described in [25]. Sam68 was purchased from Santa Cruz. Src specific (2.17) antibody was a generous gift of Dr S. Parsons (University of Virginia, VA, USA). c-Yes and β-catenin antibodies were from BD Transduction Laboratories. c-Src (GD11), FAK antibodies were from Upstate. Lyn, E-cadherin (H-108) antibodies were from Santa Cruz. Phospho-Src (Y418) and phospho-FAK (Y861) were from Biosource. Transferrin Receptor (TR) antibody was from Zymed. α-pTyr 4G10 and anti-αtubulin were a gift from P. Mangeat and N. Morin respectively (CRBM, Montpellier). Alexa Fluor 488 dye conjugated goat α-mouse or goat α-rabbit and Alexa Fluor 594 dye conjugated goat anti-Mouse or goat anti-rabbit were from Alexa. siRNA SmartPools (c-Src, c-Yes, Lyn, FAK) were from Dharmacon. c-Yes shRNA sequence was: sense: 3′-cagaaucccuccaugaauuuu; antisense: 5′-aauucauggagggauucuguu. A double stranded version containing the loop sequence ctcaagaga was synthesised (Proligo, France) and inserted in pSilencer2.1-U6 neo (Ambion). A pSilencer control shRNA (Ambion) was used. An shRNA-resistant version of c-Yes cDNA (called R-Yes) was generated by site-directed mutagenesis (QuickChange II XL-Stratagen) of full-length human c-Yes cDNA and inserted into pcDNA.1DV5HisTopo (Invitrogen). Primers used were: 5′-ccctcagggctgtccagagtcgctgcacgaattgatgaatc-3′ and 5′-gattcatcaattcgtgcagcgactctggacagccctgaggg-3′. To generate a “kinase-dead” version of R-Yes (R-Yes-K^−^), Lysine 308 was mutated to Methionine. Primers used were: 5′-gcaatcaaaacactaatgccaggtacaatgatgccagaagc-3′ and reverse: 5′-gcttctggcatcattgtacctggcattagtgttttgattgc-3′. Human cDNA for Src, R-Yes and R-Yes-K^−^ were subcloned into pBABE puro-retroviral vector.

### 2.2. Cell culture, transfections and infections

HT29, SW620, SW480, HCT116 and Colo205 cell lines were obtained from ATCC and grown at 37°C and 5% CO_2_ in RPMI containing 10% fetal calf serum (Sigma). Transfections of siRNAs into HT29 cells were carried out using 3 µl Lipofectamine 2000 reagent plus 3 µl of SmartPool solution at 20 µM. To generate stable shRNA c-Yes clones, pSilencer-Yes shRNA or sh-control Ambion construct (srb1) were transfected into HT29 cells and neomycin-resistant colonies were amplified (400 µg/ml). To generate stable shRNA Src pools, HT29 cells were transduced with Sigma MISSION™ shRNA vectors for Src (SHVRSC-TRCN0000038149; MOI 100 or 300) or a non-target scramble control sequence (srb2) (SHC002V; MOI 100). For restoration experiments, shRNA Yes pool was transduced with c- Src, R-Yes or R-Yes-K^−^ constructs by retroviral infection as in [26].

### 2.3 Cell growth and migration

For anchorage-independent cell growth, 0.67% agar in medium was layered on the bottom of a 12 wells plate and 2 000 cells/well were seeded on the top of this layer in 0.33% agar-medium. After 18–21 days colonies having >50 cells were scored positive. Cell migration assays were performed in Boyden chambers (BD Bioscience) using 50,000 cells for 2 h.

### 2.4. Biochemistry

Cell lysate, immunoprecipitation, Western-blotting and in vitro kinase assays for SFKs were preformed as described in [27]. Membrane/cytoplasm fractionations were done using Subcellular Protein Fractionation Kit according to the manufacturer's instructions (Pierce, ThermoScientific). AnnexinV/PI labelling and caspase assays were performed as follows: Cells were washed with PBS without calcium/magnesium and once with annexin V buffer (10 mM Hepes, 2.5 mM CaCl_2_, 140 mM NaCl. 20 µl annexin V-Alexa fluor 488 conjugate (Invitrogen) and 10 µl propidium iodide (PI) at 2.5 µg/ml were added and incubated 15 min in the dark. Readings were performed with an XL/MCL flow cytometer (Beckman Coulter). Alexa fluor 488 and PI fluorescences were collected (520 nm and 630 nm filters).

### 2.5. RNA extractions and RT-QPCR

HT29 were scrapped in 1 ml TRIZOL® (Invitrogen P/N 15596-026) and total RNA was extracted according to manufacturer's protocol. Quality of RNA was estimated with the ratio 28S/18S RNA in the sample using Agilent Bioanalyser 2100. Reverse transcription was performed with the High capacity cDNA reverse transcription kit (Applied Biosystems P/N 4374966). PCR was performed using an Applied Biosystem 7300 Real time PCR system and TaqMan Assay (Applied Biosystem): Yes (Hs00736972_m1), Src (Hs00178494_m1), VEGF-A (Hs00900055_m1), EPHB3 (Hs00177903_m1), 18s (Hs99999901_s1) and GAPDH (Hs99999905_m1). RNA 18S and GAPDH endogenous controls were used for quantification.

### 2.6. Mouse xenograft assays

Murine subcutaneous tumor growth were performed as follows: 1×10^7^ cells viable cells were inoculated subcutaneously (into the flank) to 6–8 week old female SCID mice (Charles River). Tumors size measured with calipers. At days 21 or 30, mice were sacrificed, subcutaneous tumors were removed and processed for analysis.

Murine liver invasion assays were performed as follows: female SCID mice were purchased from Charles River and maintained in a specific pathogen-free environment. 1.5×10^6^ were inoculated into the spleen of anaesthetized SCID mice. The spleen was removed after 3 minutes following the end of cell injection. Mice were monitored daily for up to 30 days. At day 30, mice were sacrificed, livers were removed and processed for histo-pathological analysis by a trained pathologist. Micrometastases (<2 mm) or metastases (>2 mm) were quantified by the relative surface covered by invading cells. Micrometastases were scored as minimal (one single point), +, ++ or +++. Metastases were scored as +, ++ or +++. To obtain a weighed average of the level of invasion observed in each group of three to four mice, a numerical score was attributed to each relative value: minimal = 0.5, + = 1.0, ++ = 2.0 and +++ = 3.0. These scores were multiplied by the number of mice in one group presenting the score and the total of added values divided by the number of mice in one group. The “metastatic index” is calculated for each group as:
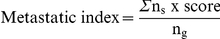
where n_s_ is the number of mice in one group presenting a particular score in the scale and n_g_ is the total number of mice in one group. All experiments on live animals were performed in accordance with institutional and national guidelines and regulations. Protocols have been approved by the Institut de Recherches Servier Ethics Committee (ID for both protocols: EFFIXENOSOU; Approval for both protocols on 26 January 2009).

## Supporting Information

Figure S1 **SFK expression in cells expressing indicated shRNA.**
(TIF)

Figure S2 **Transient c-Yes but not c-Src knock-down results in cell clusters.**
A. c-Src and c-Yes level and activity in cells transfected with indicated siRNA.
B. c-Src, but not c-Yes knock-down inhibits FAK phosphorylation at Tyr861.
C. Transient c-Yes knock-down induces cell-cell clustering.
(TIF)

Figure S3 **Fak and Lyn knock-down does not induce cell clusters.**
(TIF)

Figure S4 **Confocal microscopy analysis of E-cadherin and β-catenin localisation in c-Yes knock-down cells.**
(TIF)

Figure S5 **Yes knock-down modify cell cycle and increase apoptosis.**
A. c-Yes knock-down increase SubG1 and G2M cell cycle fractions.
B. c-Yes knock-down increase cell doubling time (measure in hours by BrDu incorporation). C. c-Yes knock-down increase caspase activity (in fluorescence units).
(TIF)

Figure S6 **c-Src and c-Yes levels in samples 17 or 31 days after implantation in mice of HT29 cells expressing indicated shRNA.**
(TIF)

Figure S7 **R-Yes, but not R-Yes-K^-^ construct restores β-catenin** **localisation in HT29 c-Yes knock-down cells.**
(TIF)
