# Supplementary figures and images for: Specific Oncogenic Activity of the Src-Family Tyrosine Kinase c-Yes in Colon Carcinoma Cells
Source: PLoS One. 2011 Feb 24;6(2):e17237. doi: 10.1371/journal.pone.0017237 (PMC3044743; doi:10.1371/journal.pone.0017237)

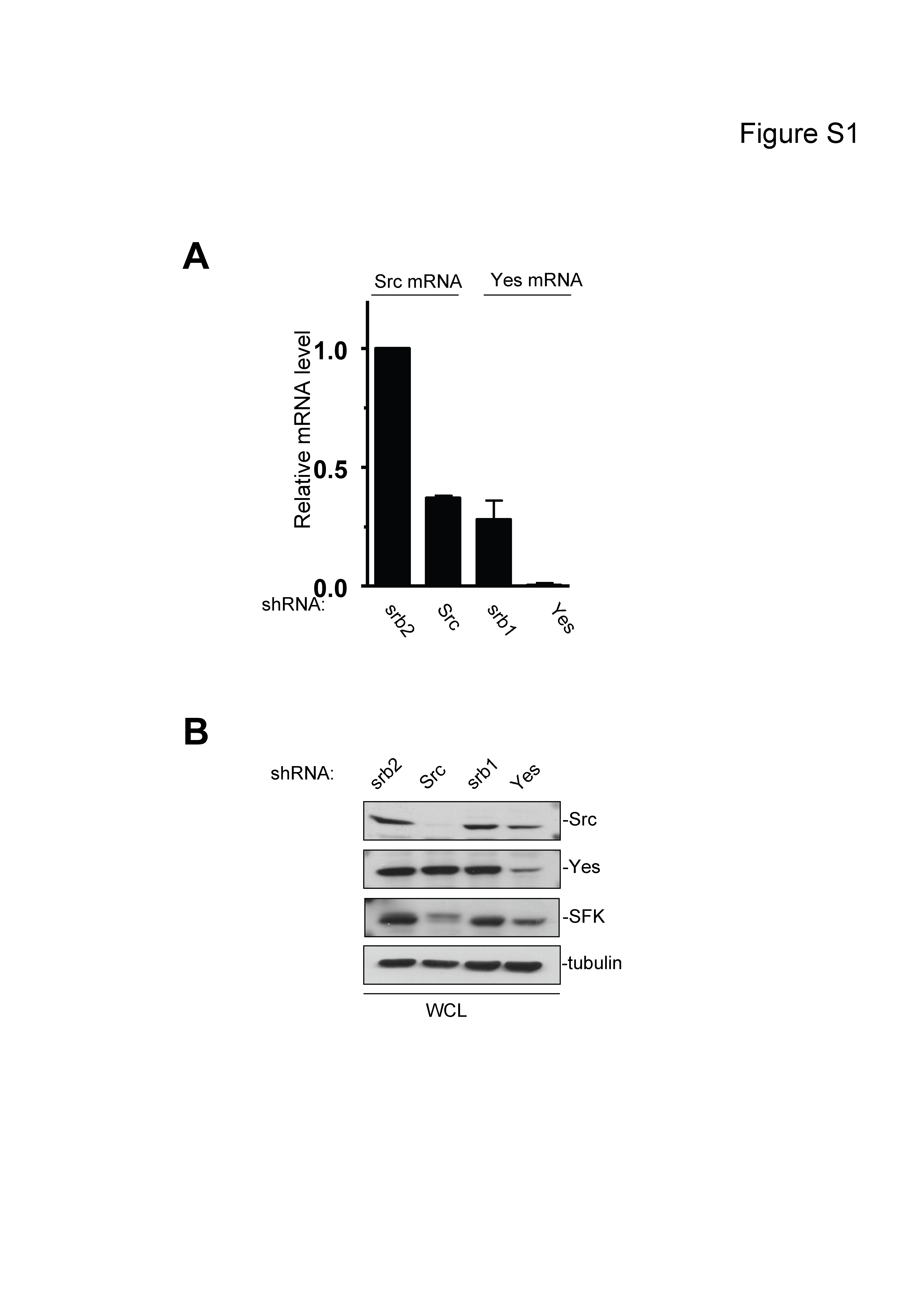

Supplement: Figure S1 — SFK expression in cells expressing indicated shRNA. (TIF) [file pone.0017237.s001.tif]

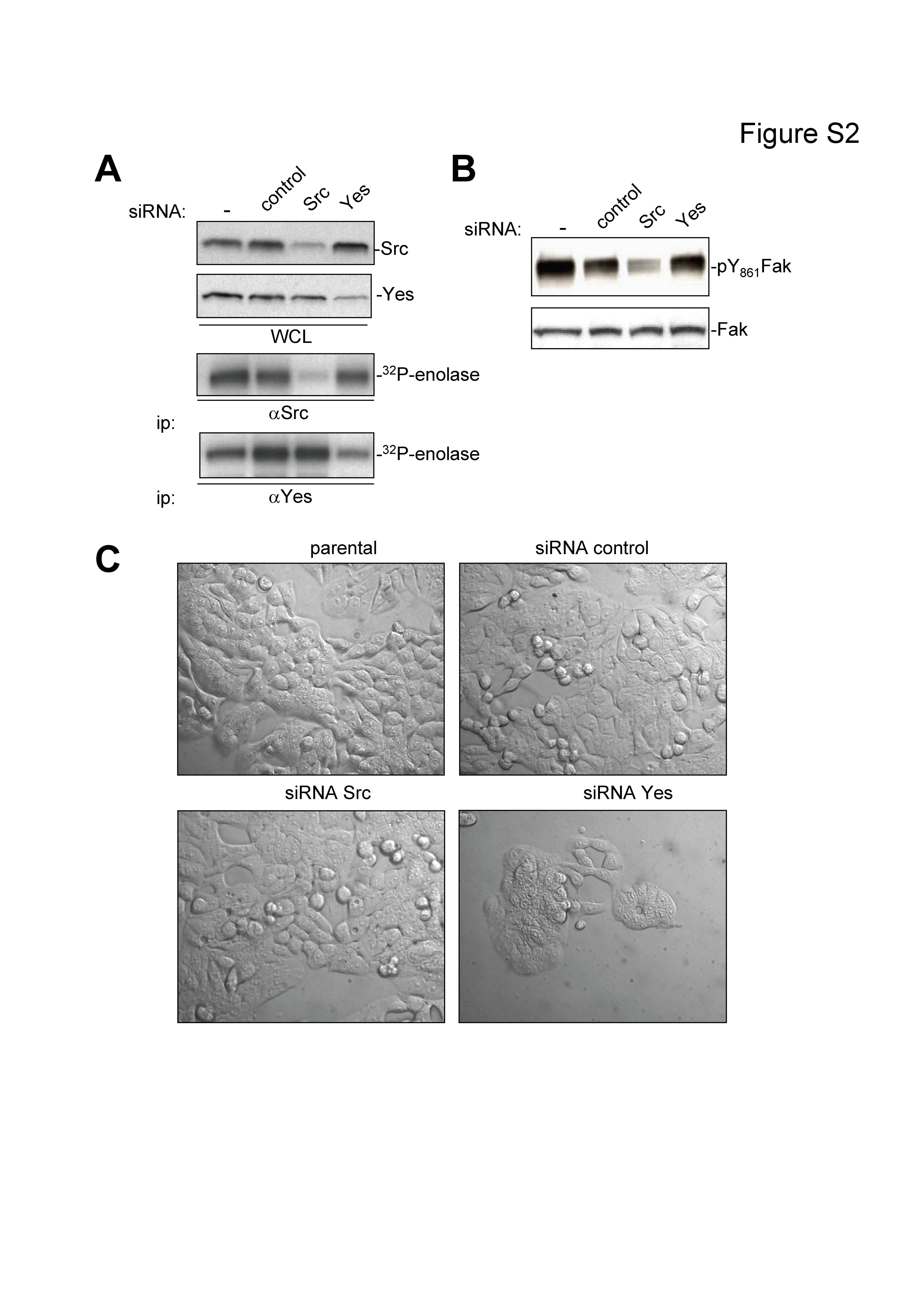

Supplement: Figure S2 — Transient c-Yes but not c-Src knock-down results in cell clusters. A. c-Src and c-Yes level and activity in cells transfected with indicated siRNA. B. c-Src, but not c-Yes knock-down inhibits FAK phosphorylation at Tyr861. C. Transient c-Yes knock-down induces cell-cell clustering. (TIF) [file pone.0017237.s002.tif]

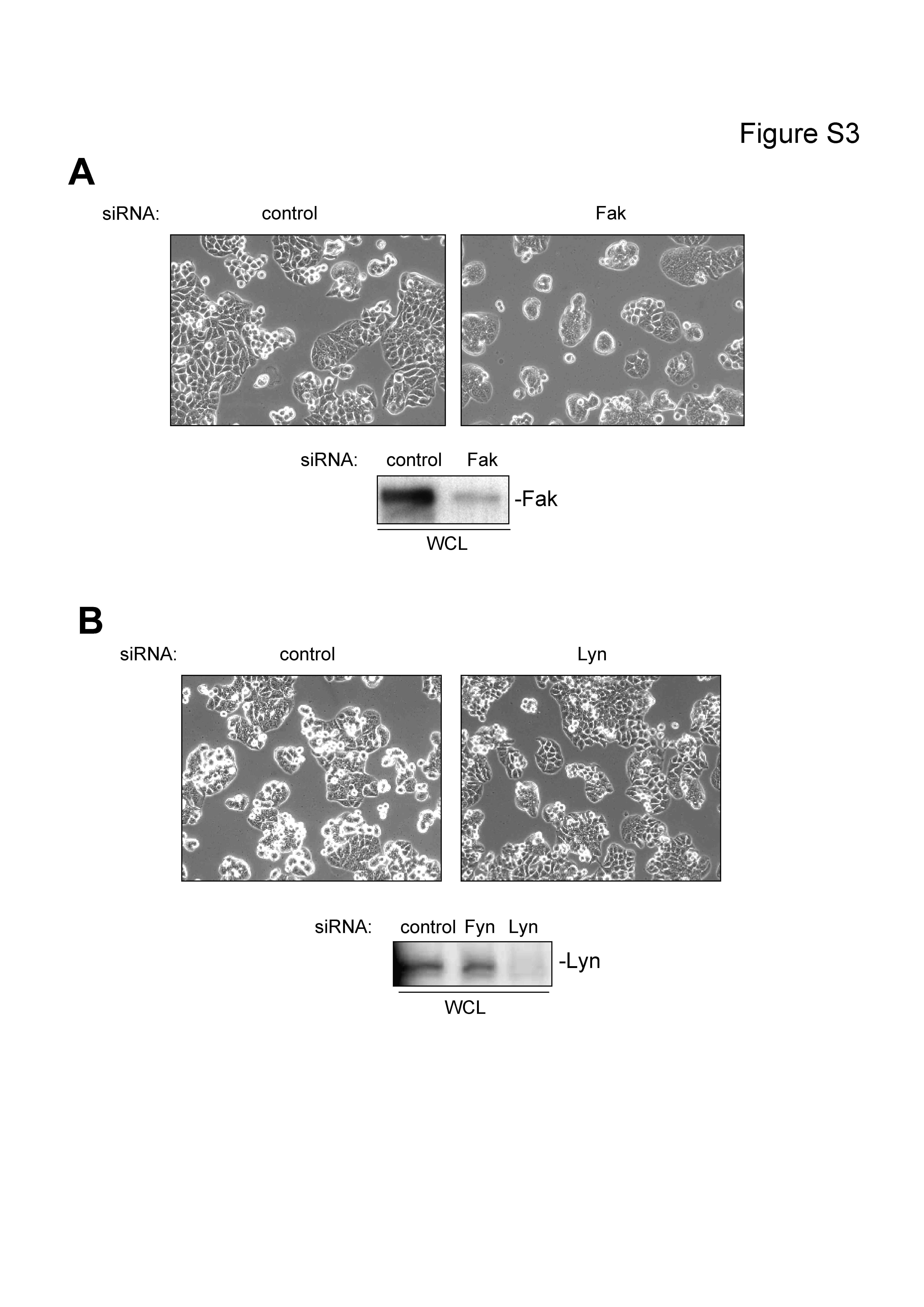

Supplement: Figure S3 — Fak and Lyn knock-down does not induce cell clusters. (TIF) [file pone.0017237.s003.tif]

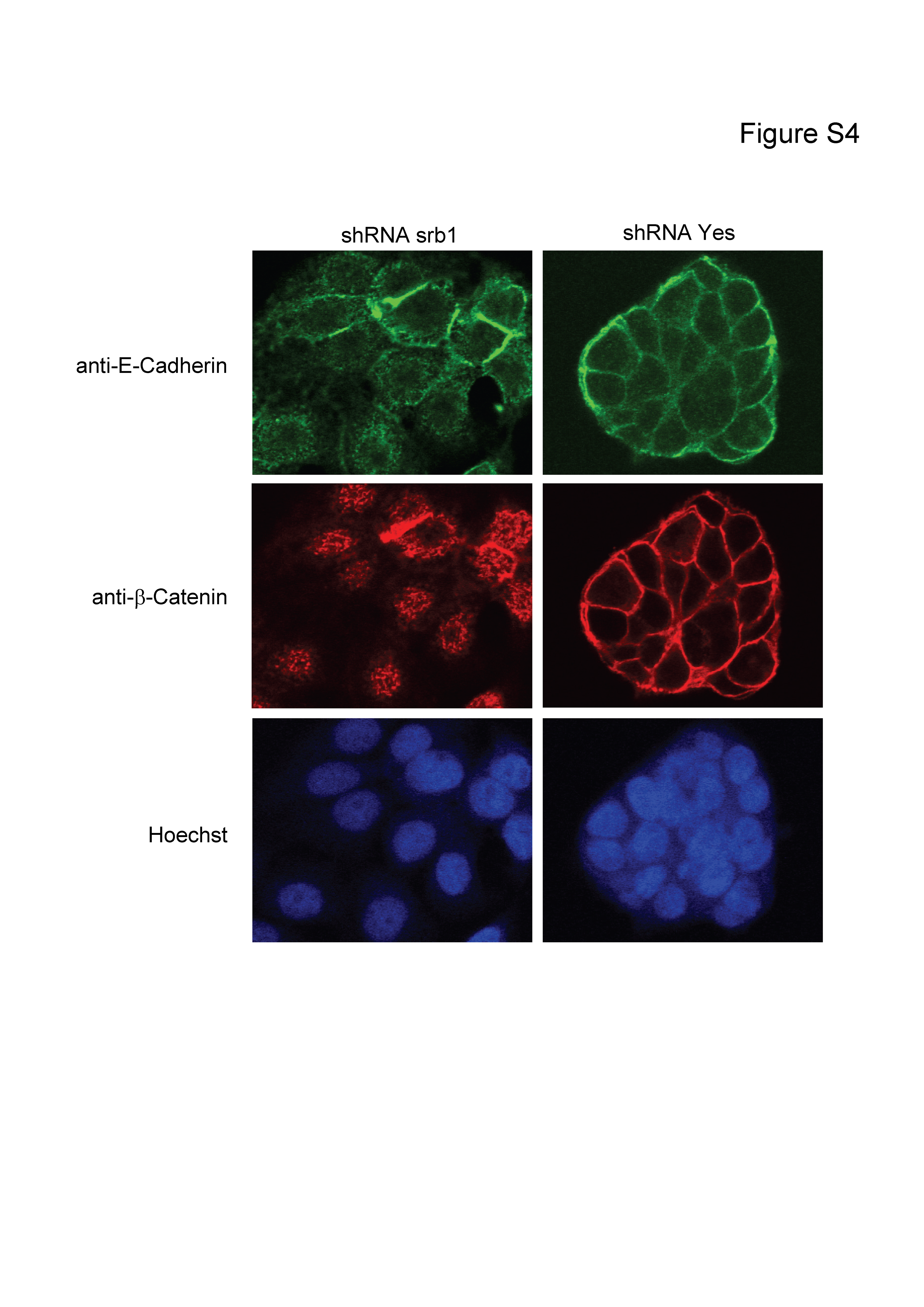

Supplement: Figure S4 — Confocal microscopy analysis of E-cadherin and β-catenin localisation in c-Yes knock-down cells. (TIF) [file pone.0017237.s004.tif]

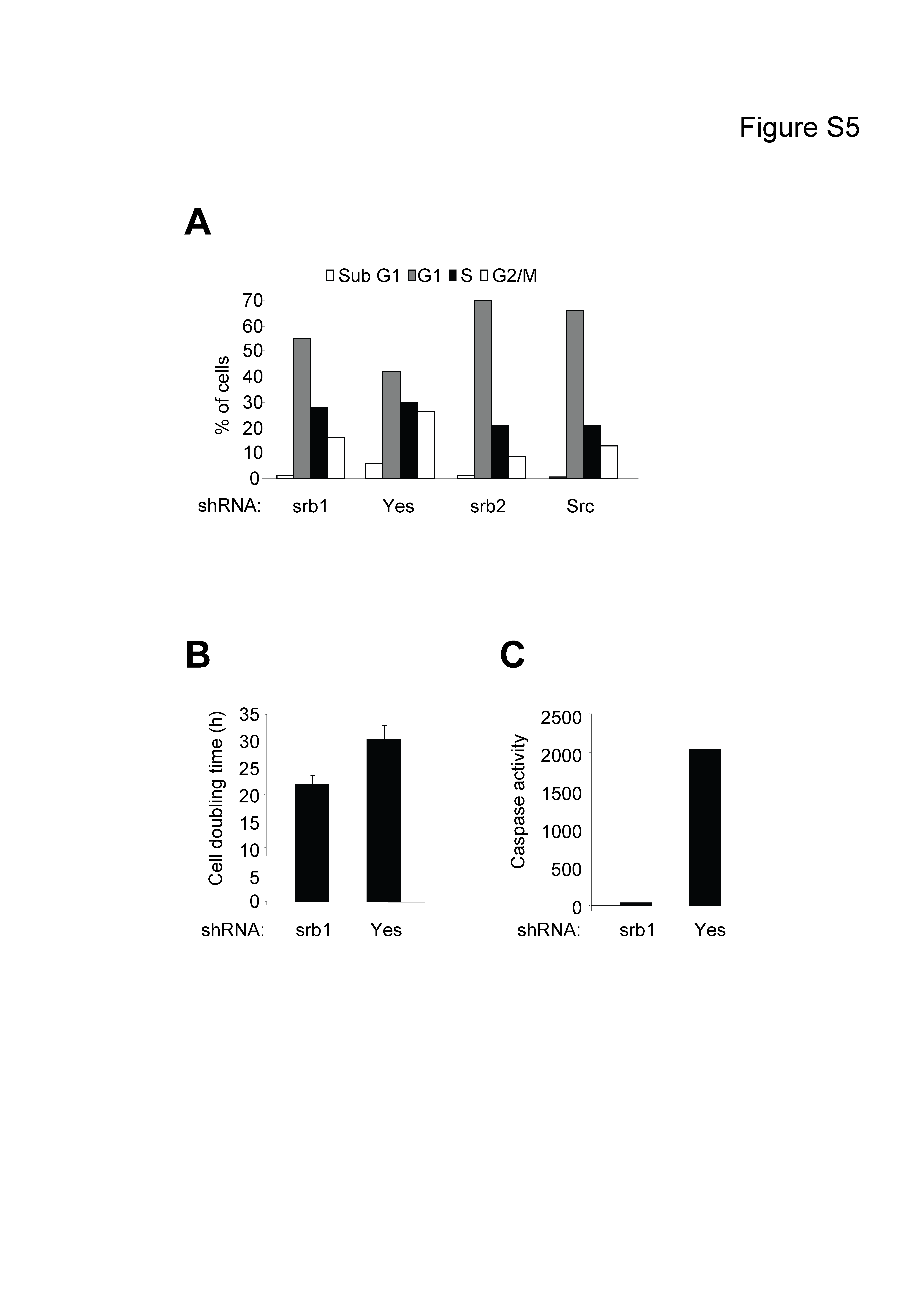

Supplement: Figure S5 — Yes knock-down modify cell cycle and increase apoptosis. A. c-Yes knock-down increase SubG1 and G2M cell cycle fractions. B. c-Yes knock-down increase cell doubling time (measure in hours by BrDu incorporation). C. c-Yes knock-down increase caspase activity (in fluorescence units). (TIF) [file pone.0017237.s005.tif]

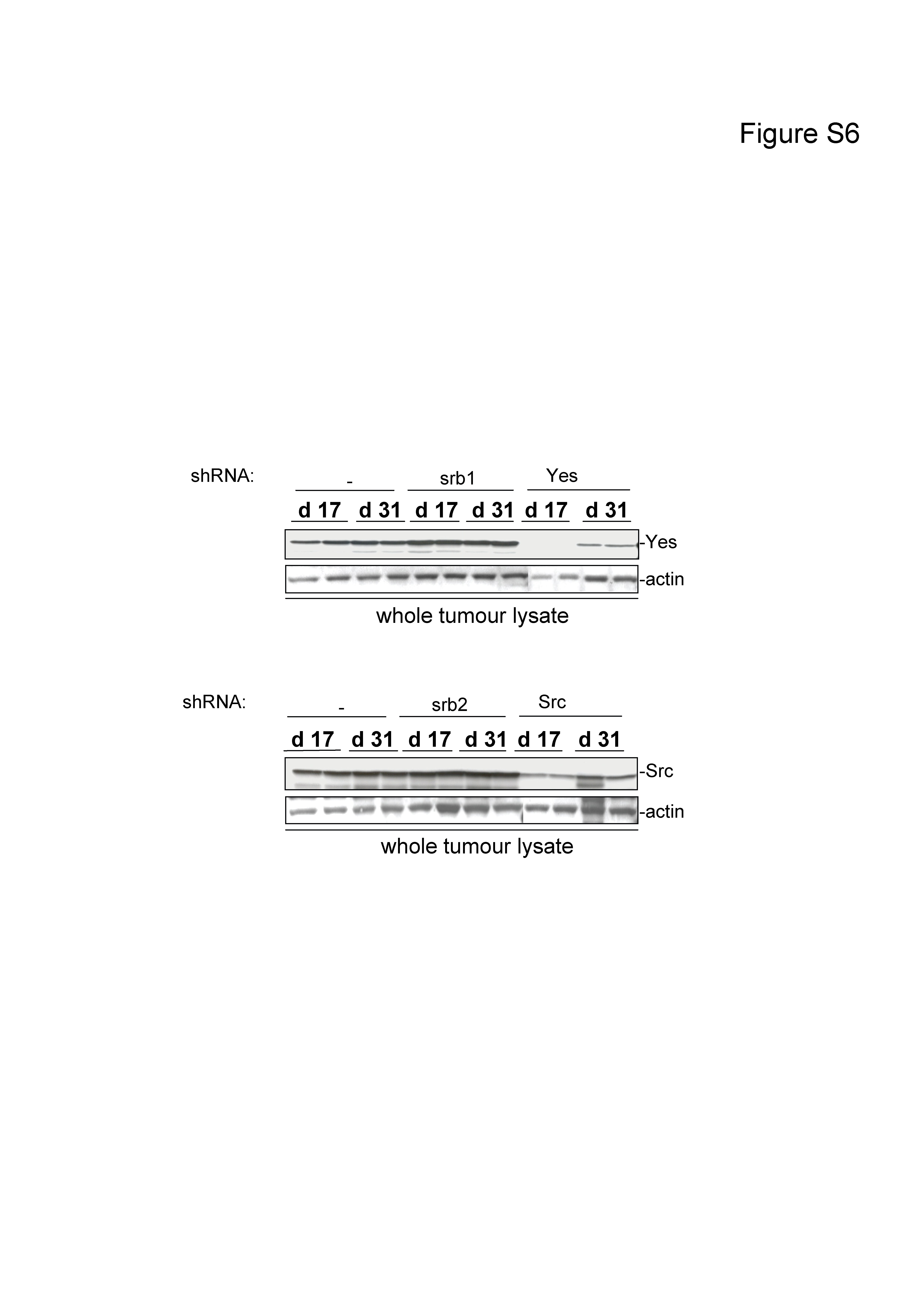

Supplement: Figure S6 — c-Src and c-Yes levels in samples 17 or 31 days after implantation in mice of HT29 cells expressing indicated shRNA. (TIF) [file pone.0017237.s006.tif]

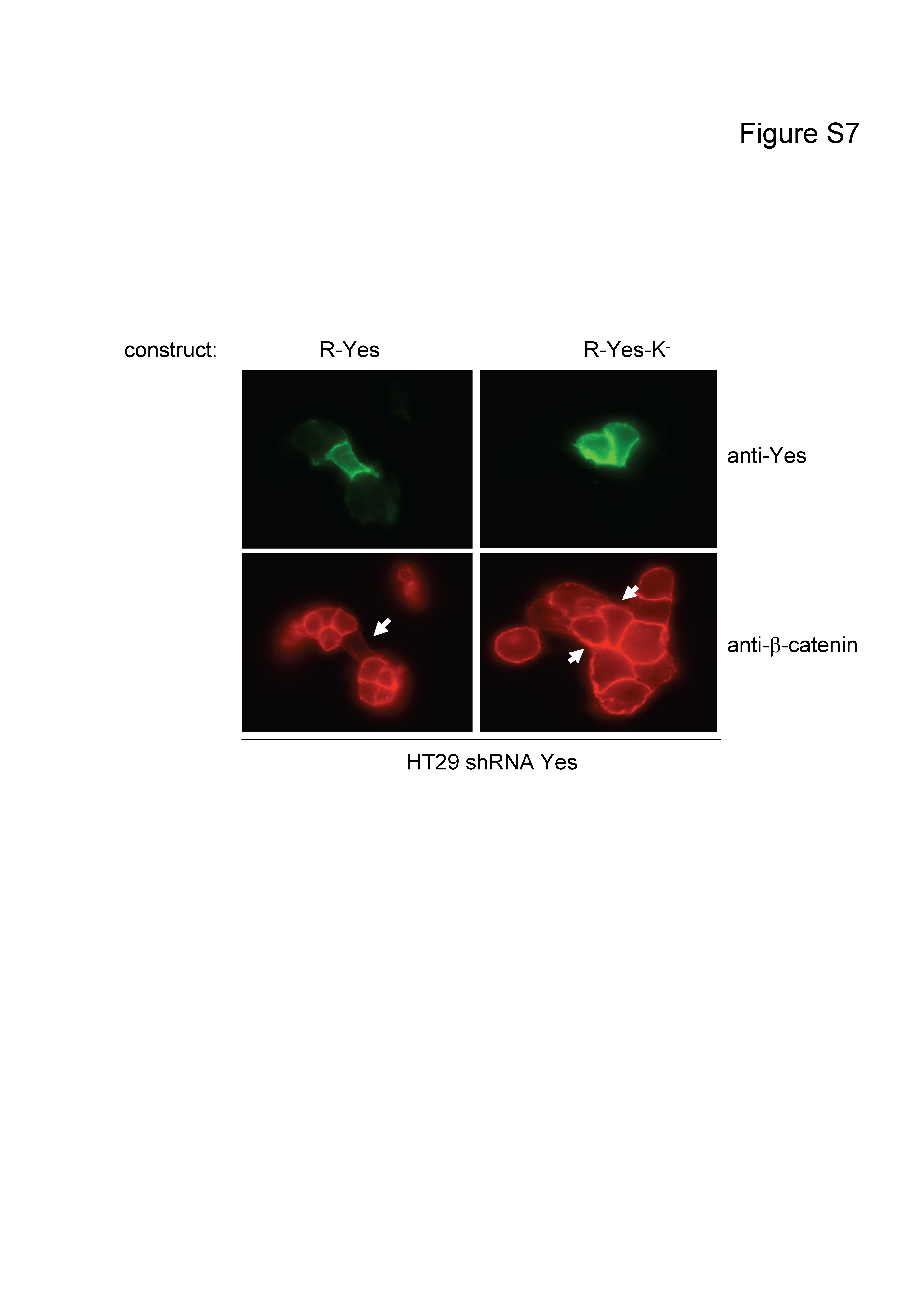

Supplement: Figure S7 — R-Yes, but not R-Yes-K- construct restores β-catenin localisation in HT29 c-Yes knock-down cells. (TIF) [file pone.0017237.s007.tif]
